# Factors associated with high job satisfaction among care workers in Swiss nursing homes – a cross sectional survey study

**DOI:** 10.1186/s12912-016-0160-8

**Published:** 2016-06-06

**Authors:** René Schwendimann, Suzanne Dhaini, Dietmar Ausserhofer, Sandra Engberg, Franziska Zúñiga

**Affiliations:** Institute of Nursing Science, University of Basel, Bernoullistr. 28, Basel, 4056 Switzerland; College of Health-Care Professions Claudiana, Lorenz-Böhlerstr. 13, Bozen, 39100 Italy; Pittsburgh University, School of Nursing, 350 Victoria Building, 3500 Victoria St, Pittsburgh, PA 15261 USA

**Keywords:** Nursing homes, Care workers, Job satisfaction, Work environment, Leadership

## Abstract

**Background:**

While the relationship between nurses’ job satisfaction and their work in hospital environments is well known, it remains unclear, which factors are most influential in the nursing home setting. The purpose of this study was to describe job satisfaction among care workers in Swiss nursing homes and to examine its associations with work environment factors, work stressors, and health issues.

**Methods:**

This cross-sectional study used data from a representative national sample of 162 Swiss nursing homes including 4,145 care workers from all educational levels (registered nurses, licensed practical nurses, nursing assistants and aides). Care worker-reported job satisfaction was measured with a single item. Explanatory variables were assessed with established scales, as e.g. the Practice Environment Scale – Nursing Work Index. Generalized Estimating Equation (GEE) models were used to examine factors related to job satisfaction.

**Results:**

Overall, 36.2 % of respondents reported high satisfaction with their workplace, while another 50.4 % were rather satisfied. Factors significantly associated with high job satisfaction were supportive leadership (OR = 3.76), better teamwork and resident safety climate (OR = 2.60), a resonant nursing home administrator (OR = 2.30), adequate staffing resources (OR = 1.40), fewer workplace conflicts (OR = .61), less sense of depletion after work (OR = .88), and fewer physical health problems (OR = .91).

**Conclusions:**

The quality of nursing home leadership–at both the unit supervisor and the executive administrator level–was strongly associated with care workers’ job satisfaction. Therefore, recruitment strategies addressing specific profiles for nursing home leaders are needed, followed by ongoing leadership training. Future studies should examine the effects of interventions designed to improve nursing home leadership and work environments on outcomes both for care staff and for residents.

## Background

In long-term care facilities, ongoing societal and demographic changes are increasing both the number of care-dependent older people and the demand for professional nursing care [1, 2]. For example, in Switzerland it is estimated that the population of nursing staff will need to grow by 30% by 2020 to cover stationary long-term care needs [3]. However, nursing homes’ attempts to meet the rising demand are impeded by considerable annual turnover rates among care workers [4]. Since higher job satisfaction is closely linked to less intention to leave and lower turnover [5–11], the identification of modifiable factors associated with job satisfaction might support nursing home managers in reducing turnover and maintaining high care quality. In both hospital- and long-term care-based empirical studies, several such factors have already been found. These link health personnel job satisfaction with working conditions and work environment, job stress, role conflict and ambiguity, role perception and content, and organizational and role commitment [12–14]. To date, however, few nursing home studies have examined a wider combination of organizational factors related to job satisfaction [14].

Overall, nursing home care workers with diverse educational backgrounds report moderate to high job satisfaction [15–18]. In the U.S., data from the 2004 National Nursing Home Survey indicated that 82 % of nursing assistants were satisfied or extremely satisfied with their jobs [19]; in Sweden, 76 % of nursing assistants reported moderate or high general job satisfaction [20]. In a recent concept analysis, job satisfaction was defined as “*an affective reaction to a job that results from the incumbent’s comparison of actual outcomes with those that are desired, expected and deserved”* (p 130) [21]. Accordingly, the complex phenomenon of nurses’ job satisfaction depends upon their sense of personal accomplishment, their personal expectations, and the nature of their jobs [12]. Along with broad differences in these variables among care workers, methodological differences in measuring them can lead to broad variations in care workers’ job satisfaction and its antecedents, not only between countries but also between settings and professional groups. A recent review compared results concerning personal and organizational antecedents of job satisfaction between nurses in hospitals and care aides in residential long-term care [14]. In the latter group, observed variations included a higher valuation of workload and lower valuation of coworker support regarding job satisfaction. Although the question remains open whether this reflects differences in educational backgrounds or in settings, it shows that desired, expected or deserved outcomes vary considerably among healthcare professionals, and that a context-specific approach is needed to identify relevant antecedents.

Both personal and organizational factors have been examined as possible antecedents of job satisfaction. In nursing home studies, results concerning individual (e.g., age) or facility characteristics (e.g., bed count) show either no relationships or equivocal ones, while organizational factors are often positively associated with job satisfaction [14, 22]. Higher job satisfaction among nursing home care staff is related to the opportunity to provide high-quality, person-centered care [13, 20, 23], to effective leadership [24] and teamwork [25], and to resident satisfaction [26]. Lower job satisfaction correlates with shortages of qualified personnel [27], inadequate supervision [28], lack of cooperation [28, 29], health complaints, and absence due to illness [20, 30, 31]. At the organizational level, along with a lack of opportunities for advancement and professional growth, insufficient compensation appears to contribute strongly to job dissatisfaction [23–25, 27], while greater job autonomy, job control, and involvement in decision-making are all associated with higher satisfaction [32–36].

Care workers with lower job satisfaction not only have a high probability of leaving their job [5, 6], but also show higher rates of absenteeism and emotional exhaustion or burnout [12]. Therefore, in addition to supporting staff stability, improvement of factors related to higher job satisfaction in nursing homes might also advance the well-being of care workers, and by extension, of the residents who depend on them.

In Switzerland, the nursing home sector employs over 120,000 persons in 1,558 facilities with over 92,000 beds (median size: 59 beds) [37]. Nursing homes have public, private or mixed ownership and offer services ranging from adult daycare and post-acute care (including rehabilitation) to dementia care and long-term care in home-like environments [38]. Roughly 30 % of these facilities’ care workers are registered nurses, complemented by licensed nurses (21 %, 3 years’ education) and nurse aides (21 %: 1–2 year’ education; 28 %: training on the job). In view of the growing number of older people in Switzerland, the pressing need for more care services, and the difficulties replacing staff members–especially registered nurses–in nursing homes [38], nursing home administrators face increasing challenges to uphold the quality of care and service. The identification of factors that contribute to nursing home care workers’ job satisfaction is highly relevant to staff retention and ultimately to safe care.

### Literature gap

Although multiple nursing home studies have examined job satisfaction and its antecedents, it remains unclear which factors are most influential regarding high job satisfaction in this setting. While previous studies focused on isolated influencing factors, they lacked a comprehensive exploration of multiple organizational factors, particularly including aspects and combinations of work environment and care workers’ health simultaneously.

**Fig. 1:**
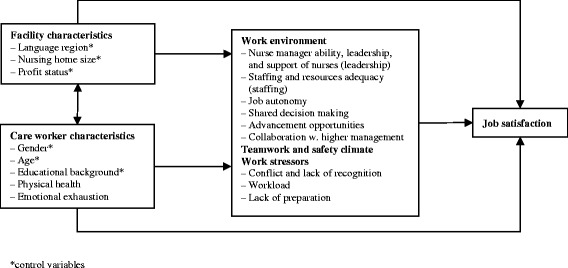
Nursing home and care worker characteristics and workplace factors related to job satisfaction

For the current study, then, we approached job satisfaction as an outcome determined by a combination of organizational and personal factors–to which we added care workers’ health complaints. Controlling for facility and care worker characteristics, we focused on modifiable factors including the work environment (e.g., leadership), work stressors and the teamwork and safety climate (see Fig. 1).

## Methods

### Study aims

The goals of this study are 1) to measure job satisfaction among Swiss nursing home healthcare workers, and 2) to examine how work environment, work stressors, and care workers’ health complaints are associated with job satisfaction in nursing homes.

### Design and sample

This study utilizes data from the *Swiss Nursing Homes Human Resources Project* (SHURP), a cross-sectional multi-center study using a random sample of 163 officially listed nursing homes across Switzerland, stratified according to language region (German, French, or Italian) and facility size (small: 20–49 beds; medium: 40–99 beds; large: 100 or more beds). Residential homes and hospices were excluded. In the participating nursing homes, only care workers who engaged in direct care and were employed a minimum of 8 h per week were surveyed, resulting in a final sample of 5,323 individuals. The SHURP study’s sampling and survey methods are described elsewhere in greater detail [39]. To address the objectives of this sub-study, we excluded persons in leadership positions (e.g., unit and department managers), leaving a sub-sample of 4,145 care workers from 162 nursing homes.

### Variables and measurements

Socio-demographic and professional data on care workers, including their perceptions of their work environment, work stressors, and health complaints, were collected using a structured survey questionnaire [39]. Facility characteristics were assessed via a questionnaire completed by the nursing home administrators. Both questionnaires were translated into German, French, and Italian. Items were verified against their original language versions by comparison with back translations. The care worker questionnaire’s items and scales were tested for their relevance in consultation with gerontological experts. Their content validity (item content validity index (I-CVI) or scale content validity index (S-CVI)) was confirmed, and all items were pre-tested for comprehensibility in end-user focus groups. Further information related to the development and validity testing of the questionnaire are described elsewhere [39].

#### Outcome variable

Care worker job satisfaction was measured using a single item: “How satisfied are you overall with your current job in this nursing home?“Respondents rated their satisfaction on a 4-point Likert-type scale, ranging from 1 (*strongly dissatisfied*) to 4 (*strongly satisfied*). To focus our analysis on the most satisfied respondents, we dichotomized the outcome variable as follows: 1 = strongly satisfied vs. 2 = rather satisfied, rather dissatisfied, or strongly dissatisfied. The single item approach reflects job satisfaction as a whole with high reliability and validity [40], and has been used successfully in previous hospital and nursing home studies [19, 26, 41, 42].

#### Explanatory variables

The independent variables of interest are presented in Table 1. They include assessments of work environment factors (via the Practice Environment Scale–Nursing Work Index (PES–NWI)), of teamwork and safety climate (via the Safety Attitude Questionnaire (SAQ)), and of workplace stressors (via the Health Professions Stress Inventory (HPSI)). Care workers’ health complaints were assessed using five items from the Swiss Health Survey; ratings of “feeling depleted from work” were gathered via a single item from the Maslach Burnout Inventory. Further details on items and measurement levels are described in Table 1.

**Table 1:** Description of independent variables assessing the work environment, work stressors and care workers’ health complaints

Variable Name	Description	Measurement
Work environment		
Leadership	5-item subscale “*Nurse manager ability, leadership, and support of care workers”* of the Practice Environment Scale-Nursing Work Index (PES-NWI) [60], assessing support by direct supervisors, their competency, back-up in decision making, praise and recognition given, and the use of mistakes as learning opportunities and not criticism	4-point Likert-type scale from 1 = strongly disagree to 4 = strongly agree Cronbach’s α = .84
Staffing and resource adequacy	3-item subscale “*Staffing and resources adequacy*” of the PES-NWI [60], assessing whether there was enough time and opportunity to discuss resident care problems, enough qualified personnel to provide quality resident care, and enough staff to perform all necessary tasks	4-point Likert-type scale from 1 = strongly disagree to 4 = strongly agree Cronbach’s α = .74
Job autonomy	Single item (Investigator developed), assessing whether care workers decide autonomously how to perform their work	4-point Likert-type scale from 1 = strongly disagree to 4 = strongly agree
Shared decision making	Single item of the PES-NWI [60], assessing opportunities for care workers to participate in nursing home policy decisions (e.g., about resident care or work organization)	Idem
Advancement opportunities	Single item of the PES-NWI [60], assessing opportunities for professional advancement (e.g., continuing education opportunities, special tasks within the team/in the nursing home)	Idem
Teamwork and safety climate	Combination of two subscales of the Safety Attitude Questionnaire (SAQ) [61]. Based on confirmatory factor analysis, the original two subscales for *Teamwork* and *Safety Climate* could not be confirmed. Three items with low item discrimination (corrected item-scale correlation < 0.4) were removed. This resulted in one 10-item single factor for *Teamwork and Safety Climate,* assessing, e.g., the opportunity to speak up or to ask questions when something is not understood, the extent to which other team members provide assistance when needed, the opportunity to discuss errors and to learn from each other, and the reception of feedback about one’s performance.	5-point Likert-type scale from 1 = strongly disagree to 5 = strongly agree with the option “don’t know” Cronbach’s α = .89
Available director of nursing	Single item of the PES-NWI [60], assessing whether the director of nursing is available for the care staff	4-point Likert-type scale from 1 = strongly disagree to 4 = strongly agree
Resonant nursing home administrator	Single item of the PES-NWI [60], assessing whether the nursing home administrator has an “open ear” and responds to issues raised by the care staff	Idem
Work stressors		
	Of the original 30-item Health Professions Stress Inventory (HPSI) [62], 2 items were selected based on expert ratings concerning their relevance in the nursing home context. Exploratory factor analysis identified 3 factors.	5-point Likert-type scale ranging from 0 = never to 4 = very often
Conflict and lack of recognition	6-item subscale, assessing, e.g., disagreement with other health professionals concerning residents’ treatment, conflicts with supervisors, not being asked about one’s opinion concerning decisions about one’s job, and not being paid enough	Idem Cronbach’s α = .76
Workload	3-item subscale, assessing, e.g., having so much work to do that not everything can be done well and not having enough people working to perform the work well	Idem Cronbach’s α = .74
Lack of preparation	3-item subscale, assessing, e.g., lacking the training to meet residents’ needs, being afraid of making a mistake in the residents’ treatment and being overwhelmed by caring for terminally ill residents	Idem Cronbach’s α = .63
Health complaints		
Physical health	From the original Swiss Health Survey [63], 5 items on health complaints, including back pain, joint pain, tiredness, problems with sleeping, and headache were extracted to assess care workers’ self-reported physical health. We combined the 5 items to form a sum index ranging from 0 to 10 to express care workers general health condition.	3-point Likert-type scale from “1 = not at all to 3 = strongly” Cronbach’s α = .70
Depleted from work	Single item according to the Maslach Burnout Inventory [64], assessing care workers’ feelings of being depleted at the end of a working day	7-point Likert-type scale from "0 = never to 6 = daily"

#### Control variables

*Care worker* and *nursing home characteristics* were used as control variables. *Care worker characteristics* included age and educational level (registered nurses with diploma or higher degrees, licensed practical nurses with associate degrees, and nursing assistants/nursing aides with certified education or informal in-service training). N*ursing home characteristics* included facility size, ownership status (public, private-public subsidized, and private nursing homes), and language region.

### Data collection

The SHURP survey was administered from May 2012 until April 2013. All nursing home administrators gave informed consent for their facilities’ participation and forwarded the questionnaires and return envelope packages to their care workers. Care workers individually completed and returned the questionnaires to the study cnter.

### Data analyses

Descriptive statistics (frequencies, percentages, means and standard deviations) were used to determine the prevalence of strong job satisfaction, and to summarize data on nursing home and care worker characteristics, as well as on work environment, work stressors and care workers’ health complaints. To examine the independent variables’ relationships with care workers’ job satisfaction (dichotomized as 1 = strongly satisfied vs. 2 = rather satisfied, rather dissatisfied, or strongly dissatisfied), we used logistic regression with generalized estimating equation (GEE) modeling, controlling for care workers being nested within facilities and units (Intra Class Coefficient (ICC 1) for job satisfaction: facility level: 0.07; unit level: 0.10). The model was set to control for care worker characteristics (age, educational level) and facility characteristics (size, ownership status, language region). Our analyses tested both unadjusted and adjusted models. As several variables (job autonomy, shared decision making, advancement opportunities, and collaboration with higher management) yielded left-skewed distributions, they were dichotomized accordingly for the analysis (1 = strongly agree/agree vs. 2 = disagree or strongly disagree). Multicollinearity among the independent variables was determined with the Variance Inflation Factor (VIF). All variables produced VIF outcomes below the threshold of 5 [43] and were retained for the analyses. The GEE was run with listwise deletion of missing cases. The analysis was repeated using a GEE model employing multiple imputation: all variables showed similar significance levels to the first model. A *p*-level of < .05 was considered significant. All analyses were performed using IBM^©^ SPSS^©^ Statistics for Windows^©^, Version 21.0 software (Armonk, NY: IBM Corp.).

## Results

### Sample characteristics and care workers’ job satisfaction

The final study sample consisted of 4,145 care workers from 162 nursing home facilities across Switzerland, with an overall response rate of 76.4 %. Respondents came mainly from medium sized facilities in the German-speaking region of Switzerland. Overall, care workers’ job satisfaction was high, with 36.2 % being strongly satisfied and 50.4 % rather satisfied, while 13.4 % were either rather or strongly dissatisfied. Regarding work environment factors, we observed high values for teamwork and safety climate (3.97 on a scale from 1–5) and for leadership (3.13 on a scale from 1–4), alongside low values for work stress due either to conflict and lack of recognition (.91 on a scale from 0–4) or to lack of job preparation (.68 on a scale from 0–4). In addition, relatively high proportions of respondents agreed or strongly agreed with shared decision making options (86.1 %), and with directors of nursing being available for care staff (89.6 %). All results related to facility and care worker characteristics and the examined independent variables are summarized in Table 2.

**Table 2:** Sample characteristics

	%	Mean	SD
Facility characteristics (*n* =162)			
Facility size (number of beds)			
Small (< 50)	38.9		
Medium (50–99)	46.3		
Large (≥ 100)	14.8		
Ownership status			
Public	37.0		
Private, public subsidized	26.5		
Private	36.4		
Language region			
German speaking	75.9		
French speaking	18.5		
Italian speaking	5.6		
Care worker characteristics (*n* = 4,145)			
Females (*n* = 4,105)	92.5		
Age in years (*n* = 3,750)		42.9	12.3
Educational level (*n* = 4,109)			
Registered nurse (3–4 year education)	25.8		
Licensed practical nurse (3 year education)	22.1		
Certified nurse assistant (1–2 year education)	19.2		
Nurse aide (short course, training on the job)	29.6		
Other	3.2		
Work environment			
Leadership (PES-NWI) (1–4), (*n* = 4,145)		3.13	.60
Staffing & resources adequacy (PES-NWI) (1–4), (n = 4,138)		2.82	.66
Job autonomy (^a^), (n = 4,117)	80.6		
Shared decision making(^a^), (*n* = 4,123)	86.1		
Advancement opportunities (^a^), (*n* = 4,130)	84.4		
Teamwork & safety climate (SAQ) (1–5), (*n* = 4,133)		3.97	.66
Conflict and lack of recognition (HPSI) (0–4), (*n* = 4,138)		.91	.67
Workload (HPSI) (0–4), (*n* = 4,138)		1.53	.82
Lack of preparation (HPSI) (0–4), (*n* = 4,132)		.68	.59
Resonant nursing home administrator (^a^), (*n* = 4,093)	75.7		
Available director of nursing(^a^), (*n* = 4,114)	89.6		
Care worker reported health			
Physical health (0–10), (*n* = 4,035)		3.48	2.27
Depleted from work (0–6), (*n* = 4,097)		2.88	1.82

Note: Underlined scores are preferable scores; ^a^dichotomized variables indicate proportion of respondents who agreed strongly/agreed vs. those who disagreed strongly/disagreed with item, or who rated quality of care as rather high/very high vs. rather low/very low

### Job satisfaction and workplace characteristics

Higher job satisfaction (i.e., strong satisfaction with the workplace) was significantly associated with half of the examined work environment factors. The strongest association was with leadership: the odds of high job satisfaction increased almost four-fold with each 1-point increase in leadership rating odds ratio (OR) (OR = 3.76; 95 % CI, 2.83-4.99). Similarly, the odds increased more than two-fold with each 1-point increase either in teamwork & resident safety climate (OR = 2.59; 95 % CI, 2.02-3.32), or for nursing home administrators being resonant (as opposed to not listening to care workers) (OR = 2.23; 95 % CI, 1.67-2.97). The odds of strong job satisfaction also increased significantly with staffing and resource adequacy (OR = 1.42; 95 % CI, 1.17-1.72), and decreased significantly with increases in workplace conflict (OR = .61; 95 % CI, .48-.76), being “depleted from work” (emotional exhaustion) (OR = .88; 95 % CI, .83-.93), and physical health issues (OR = .91; 95 % CI, .87-.96). For more details see Table 3.

**Table 3:** Job satisfaction and nursing home work environment characteristics*

	Job satisfaction^a^ (*n* = 3,750)		
	OR	95% CI	*p*-value
Work environment			
Leadership (PES-NWI)	3.761	2.833 − 4.993	< 0.001
Staffing & resource adequacy (PES-NWI)	1.418	1.166 − 1.724	< 0.001
Job autonomy	.788	.619 − 1.004	0.054
Shared decision making	1.351	.884 − 2.065	0.164
Advancement opportunities	1.130	.772 − 1.654	0.530
Teamwork & safety climate	2.592	2.021 − 3.323	< 0.001
Available director of nursing	1.474	.908 − 2.393	0.117
Resonant nursing home administrator	2.231	1.676 − 2.970	< 0.001
Work stressors			
Conflict and lack of recognition (HPSI)	.605	.483 − .759	< 0.001
Workload (HPSI)	.863	.737 − 1.011	0.068
Job preparation (HPSI)	.995	.829 − 1.193	0.953
Health complaints			
Physical health	.910	.866 − .955	< 0.001
Depleted from work	.877	.825− .933	< 0.001

Note: *Binary logistic regression with GEE. The model was controlled for care worker characteristics (age, educational level) and facility characteristics (size, ownership status, and language region), OR = Odds ratio, CI = Confidence interval

^a^Two groups: 1 = strongly satisfied vs. 2 = rather satisfied, dissatisfied, or strongly dissatisfied. Group 1 is reported

## Discussion

This is Switzerland’s most comprehensive study to date of associations between organizational factors, health-related issues and job satisfaction in the nursing home setting. Conducted in a representative national sample of Swiss nursing homes, it revealed that slightly over a third of care workers were strongly satisfied with their current workplace. Strong job satisfaction was significantly associated with higher ratings for supportive leadership, teamwork and safety climate, resonant nursing home administrators, and adequate staffing resources, and with lower ratings for workplace conflict and health complaints. Other work environment factors, e.g., job autonomy, the director of nursing being available to the care workers, and stress due to workload, showed no significant associations with job satisfaction.

The high overall job satisfaction ratings of care workers in Swiss nursing homes concur not only with previous findings in nursing home [9] and acute-care settings [41], but also with those derived from research in other sectors [44, 45]. While previous researchers dichotomized their data to distinguish positive job satisfaction ratings from negative [9, 19, 26], we focused exclusively on highly satisfied care workers. By examining this group’s data, we aimed to identify factors separating the average or good nursing home workplaces from the excellent. Using an approach employed by Forbes-Thompson and colleagues, we highlighted differences in nursing home performance by focusing on rather extreme cases (i.e., strong job satisfaction ratings only) [46].

In our adjusted regression model, three factors most significantly explained variations in the proportions of care workers reporting strong job satisfaction–nursing home leadership, teamwork and safety climate and the resonance of the nursing home administrator. In this context, links between perceptions of supportive leadership–particularly of individual leaders’ types and levels of interaction with their staff–strongly suggest that workers strongly satisfied with their jobs perceive that their leaders both support them and recognize their input. In addition, as observed elsewhere, leadership styles that treat care errors as learning opportunities rather than opportunities for criticism are more likely to develop trust and commitment among care workers [47, 48]. In former studies, a variety of leadership styles were examined concerning their relationship with job satisfaction. A high score on the PES-NWI “*Nurse manager ability, leadership, and support of care workers*” subscale requires a relationship-oriented leadership style that focuses on supporting care workers, developing their skills and recognizing their work with praise and appreciation [42]. Previous studies indicated that supportive managers or a relationship-oriented leadership style contribute to nurses` job satisfaction [18, 42, 49].

Interestingly, however, in nursing homes, depending on the stability of the staff, Havig et al. found a stronger effect for task-oriented leadership (focusing on planning work activities, clarifying roles and objectives, and monitoring performance) than for relationship-oriented leadership [42]. They suggest that different teams or situations might call for different leadership styles, and that teams whose work involves a high level of interdependence might need a more task-oriented style to allow more role clarity and less work stress. While we can confirm the value of supportive leadership in nursing homes, we cannot exclude the possible advantage of adapting one’s leadership style to the situation at hand.

Furthermore, the high resonance ratings of very satisfied workers toward their nursing home administrators imply that top nursing home leaders foster and maintain direct communication with front-line care workers, monitor their needs, and support the achievement of organizational goals in their daily operations [46, 50]. Donoghue & Castle [51] found that nursing home administrators who solicit and act upon their staff’s input have lower facility-level staff turnover–a key goal of promoting job satisfaction. Similarly, in a review of relationship-oriented management practices, Toles and Anderson [52] found that reciprocal staff and manager communication added to staff satisfaction as well as to effective resident care. On the other hand, where staff members perceive no connection to their managers, open communication about medical errors is impeded, as care workers feel ignored or blamed [52, 53]. I.e., lack of a connection with managers hinders both staff satisfaction and quality of care.

As interconnected work environment aspects, enhanced teamwork and safety climate are both associated with strong job satisfaction. Teamwork is vital for staff satisfaction–a key mechanism for staff retention and nursing stability at the facility level [54]. In theory, stability within an organization’s nursing system will enhance that organization’s equilibrium, and will positively influence nurses’ satisfaction [54]. Furthermore, both teamwork and safety climate involve support from colleagues regarding residents’ care. This, in turn, strengthens care workers’ sense of belonging to a strong team, fostering the personal attitude that “I would feel safe being a resident on this unit.” As observed in the current study, confidence in colleagues and stimulation from co-workers [23] are both related to positive perceptions of teamwork, fostering high job satisfaction.

Staffing adequacy ratings reflected workers’ personal senses of whether their units’ staff counts and skill mixes were sufficient to perform all necessary work while maintaining high care quality. Significantly linked to job satisfaction, staffing adequacy included care workers’ perceptions of whether they had the time and the opportunity to discuss resident care problems. However, Van Beek and colleagues [16] initially observed that apparently significant relationships between nurse manager reported staffing levels and staff job satisfaction disappeared when communication density was controlled for, i.e., that higher staffing alone did not increase care workers’ job satisfaction. Instead, where workplace satisfaction is concerned, the current study findings suggest that the effect of allocating a specific number of workers to a unit is secondary to those workers’ perceptions of staffing adequacy (including skill mix), and to their opportunities to communicate with one another.

The topic of workplace conflict and lack of recognition encompasses a range of stressors with the potential to impact care workers’ job satisfaction. Our findings confirm previous findings [55] that workplace conflict negatively correlates with job satisfaction. Typical stressors include disagreements between care workers and other health professionals concerning residents’ care, not being asked for input on decisions related to one’s job (e.g., assignment of residents, task scheduling), clashes with supervisors, not being permitted to use all one’s skills, and being underpaid. Our analyses linked conflict and lack of recognition significantly to job satisfaction. The subscale used included the item “not being paid enough,” a factor examined in studies associating nursing assistants’ job satisfaction strongly with wages and benefits [9, 26]. Additional stressful situations, e.g., work interruptions or input from non-health professionals on how to do one’s work [56], are not explicitly identified in the current study. Nevertheless, care workers in environments where workplace conflict is poorly managed tend not only to exhibit reduced productivity [57], but also to identify poorly with their team and to report low job satisfaction [58].

Health issues, including emotional strain (reflected in feelings of emotional exhaustion or depletion at the end of a working day) and physical symptoms of stress (such as back pain, headache, tiredness or problems with sleeping) were also inversely associated with strong job satisfaction. Our findings corroborated those of earlier studies linking low job satisfaction with emotional exhaustion [31] and physical health complaints [20] in nursing home care staff as well as with the burden of emotional stress and depression in critical care nurses [59]. These findings suggest that physical discomfort and emotional exhaustion deplete one’s energy, impairing performance, inducing low mood, depression and unpleasant feelings, and ultimately reducing job satisfaction.

### Strengths and limitations

The greatest strength of this job satisfaction study among care workers in Swiss nursing homes was its extensive dataset–the product of a large representative nursing home sample and high response rates. Additionally, the strict focus on strong job satisfaction responses allowed identification of the associations most relevant to the nursing home care workforce. However, the findings should be interpreted with caution in view of its limitations. First, as its cross-sectional design captures care workers’ job satisfaction and associated factors only at a single instant, no causal relationships can be inferred. Second, considering the complexity of a socially determined construct such as job satisfaction, the use of a single item to measure it might be disputable. Nevertheless, previous studies have successfully applied similar measures to job satisfaction, as well as to related workplace factors and perceptions [19, 26, 42]. Third, the selection of items examined in relation to job satisfaction was limited to those used in the SHURP study. Other potentially relevant factors, such as work-family conflict or the opportunity to provide person-centered care, were left unexamined. Finally, social desirability bias might have skewed the results towards the positive end, reflecting the workers’ desire to be members of a good workplace.

## Conclusions

This study revealed significant associations between strong job satisfaction in Swiss nursing home care workers and six work environment factors: nursing home leadership, teamwork and safety climate, the resonance of the nursing home administrator, workers’ perceptions of staffing adequacy, workplace conflict, and health complaints. Of these, the effectiveness of the nursing home leadership at both levels–unit supervisor and executive administrator–figured most prominently in care workers’ job satisfaction. While this finding is supported by various studies on the characteristics of effective leadership, role modeling is a complex task. Clearly, recruitment strategies addressing specific leader profiles and skills are necessary, as well as ongoing executive supervision, mentoring and support, including specific leadership training, particularly for middle management positions.

For future studies, we recommend developing and testing complex interventions necessary to develop and measure the effects of enhanced nursing home leadership competencies on care staff outcomes as well as on residents’ health and quality of care.

## Abbreviations

CI, confidence interval; GEE, generalized estimating equation; HPSI, health professions stress inventory; ICC, intra class coefficient; I-CVI, item content validity index; OR, odds ratio; PES–NWI, practice environment scale–nursing work index; SAQ, safety attitude questionnaire; S-CVI, scale content validity index; SHURP, swiss nursing homes human resources project; VIF, variance inflation factor
